# Minimally Invasive Osteosynthesis of Intraarticular Calcaneus Fracture Augmented by Femoral Head Allograft: A Retrospective Study

**DOI:** 10.7759/cureus.28684

**Published:** 2022-09-01

**Authors:** Mohamad K Moussa, Emma Vuilletet, Ali Alayane, Mohammad O Boushnak, Justine Fleurette, Nicolas Nicolas, Maurice Thiongo, Zied Missaoui, Georges Kassab

**Affiliations:** 1 Orthopedic Surgery, Grand Hôpital de l'Est Francilien-Site de Meaux, Meaux, FRA; 2 Orthopedic Surgery, Hôpital Erasme-Cliniques Universitaires de Bruxelles, Bruxelles, BEL; 3 Orthopedic Surgery, Université libre de Bruxelles, Bruxelles, BEL; 4 Orthopedic Surgery, Ambroise Paré Hospital, Paris, FRA

**Keywords:** essex-lopresti technique, calcaneus joint depression fracture, minimally invasive osteosynthesis, sanders classification, percutaneous osteosynthesis, calcaneus fracture

## Abstract

Aim: The aim of this retrospective study is to report the results of minimally invasive osteosynthesis when used for the treatment of intraarticular calcaneus fracture. This old technique is regaining popularity recently with the multiple advances added by different institutes when it is used in the management of intraarticular calcaneus fracture.

Methods: Twenty-four patients who suffered from intraarticular calcaneus fractures between 2014 and 2019 were included. Twelve of them had Sanders II fractures (group A) and 12 had Sanders III + IV fractures (group B). The mean follow-up duration was 37.5 months. The mean age at presentation was 54.23 ± 12.48 years. The skin condition at presentation was poor (blood-filled blisters) in 25% of patients equally distributed between the two groups. The mean time to surgery was 5.6 days where patients with poor skin conditions were treated lately. The technique involved percutaneous ascending proximal-to-distal pinning of the calcaneus after reduction using a 2 cm mini-incision below the lateral malleolus and augmenting the fixation with femoral head cancellous allograft. The primary outcomes variables analyzed in this study are post-operative Bohler angle, post-operative Gissane angle, American Orthopedic Foot and Ankle Society (AOFAS) ankle/hindfoot score at long-term follow-up (Excellent>95, Good 75-94, Fair 51-74, poor 0-50), and the delta angle benefit score. The secondary outcomes included post-operative complications such as infection and osteoarthritis.

Results: The radiological results showed significant improvement of Bohler angle from 6.09° ± 21.6 pre-operatively, to 31.79° ± 14.1 postoperatively with a p-value <0.001. An adequate reduction was achieved in 54.16% to 70.8% of patients. There is a trend to normalization of overcorrected fracture especially Sanders II with a mean reduction of 12,71° ± 11,88 at one year post-operatively (p=0.05). AOFAS score at the last follow-up shows 20.83% poor results (AOFAS<50), 50% fair results (AOFAS between 51-74), 16.67% good results (AOFAS 75-94), and 12.5% excellent results (AOFAS>95. The satisfaction rate was 83.3% (45.8% partially satisfied, and 37.5% fully satisfied). The incidence of superficial infection (wound inflammation and pin tract infection) was more prevalent in higher group B (40%) compared to group A (0%) with p=0.014. Other complications including osteoarthritis and varus deformity were found in 95.8% and 58.3% of patients respectively at three-year follow-up.

Conclusion: The combination of minimally invasive osteosynthesis and femoral head allograft for the treatment of intraarticular calcaneus fractures seems to give fair to good functional results. Radiological data demonstrated that when the Bohler angle is over-reduced >40°, there was a tendency to autocorrection over time. This may be due to progressive depression of the angle over time as weight bearing is authorized; however, this must be analyzed carefully due to the low number of patients who were overreduced (seven patients). Our study demonstrates that this technique has a low early complication rate (especially low infection and soft tissue problems) but carries high long-term complications such as osteoarthritis and hindfoot varus.

## Introduction

Calcaneus fractures constitute 60% of all fractures affecting tarsal bones [1]. It usually results from traumatic axial loading during a fall from height or a crush injury during motor vehicle accidents. This mechanism of injury is frequently associated with other types of fractures such as tarsal fractures, vertebral fractures, ipsilateral ankle fractures, pelvic injuries, chest injuries, and contralateral calcaneus fractures [2]. The energy of trauma can propagate through primary and secondary lines, which will dictate the type of fracture whether it will be extraarticular or intraarticular [3,4]. The comprehensive analysis of the propagation of fracture lines is important since it has implications on the treatment strategy. It is well known that the primary fracture line will divide the calcaneus into a superomedial fragment and a superolateral fragment, while the secondary fracture line to dictates the articular depression. The medial fragments are in close relation with the sus-tentaculum tali and are stabilized by strong ligaments and tendons. Thus, this makes them “constant fragments” and predisposes the lateral fragments to higher energy and further articular depression [5].

Treatment of intraarticular calcaneus fracture (IACF) deserves special attention due to its association with poor functional outcomes [3]. These fractures are responsible for a decreased heel height, increased heel width, and hindfoot varus deformity. Radiological analysis facilitates the characterization of these variables by measuring Bohler and Gissan angles on the lateral foot views, as well as the measurement of the hindfoot varus deformity on the Harris view also known as the retro tibial view [3]. On the other hand, the prognosis and treatment obscurity are evaluated based on Sander’s classification, which is analyzed by computed tomography (CT) by taking the coronal images at the widest point of the posterior facet [4]. Type I includes non-displaced intraarticular fractures, while types II, III, and IV include displaced fractures with an increasing number of fracture lines passing through the posterior articular facet [4].

Treatment of IACF is controversial [4,5]. Buckley et al. state that open reduction and internal fixation may have better functional outcomes when compared with non-operative treatment in well-selected patients especially after excluding those who have received worker’s compensation [6].

However, this type of intervention presents a significant risk of complications with an overall rate of 26%. Infection (3.9%), bone necrosis (6.8%), and soft tissue problems are all reported [7]. To better understand this risk, it is paramount to know the vascularity of the calcaneal area, which is supplied in 45% by the lateral arteries. These arteries are at the greatest risk of injury during a lateral extensile approach to the calcaneus which is the most common approach utilized in an open reduction internal fixation [8].

In this article, we emphasize the importance of minimally invasive osteosynthesis associated with bone grafting (MIO with BG) in the treatment of IACF. Furthermore, the primary objective of this study is to assess the efficacy of combined MIO and BG and their relative complications when used as a modality of treatment for IACF.

## Materials and methods

As part of a single-center retrospective study, data relative to all patients who underwent MIO with BG for IACF in our institution between 2014 and 2019 were retrieved. The only indication for this procedure is a displaced IACF according to radiographs and computed tomography. Open fractures were included. Exclusion criteria were pediatric patients, patients requiring another intervention in the same limb, and psychiatric patients. Informed consent was obtained from all the participants.

Pre-and post-operative measurements included Bohler angle, Gissane angle, and hindfoot varus deformity. Post-operative measurements included the AOFAS score, version range of motion, and sagittal range of motion of the ankle joint. Complications such as infection, osteoarthritis, hindfoot varus deformity, and the need for arthrodesis, were also collected. Data was then divided into two groups depending on Sander’s classification (Sander II vs Sander III + IV). Delta benefit in terms of Bohler and Gissane angles was also calculated. The stability of reduction at three-year follow-up in terms of Bohler angle, Gissane angles, and hind foot varus were also calculated. AOFAS score was considered excellent when AOFAS>95, good if between 75-94, fair if between 51-74, and poor if between 0-50. The primary outcome variables analyzed in this study are the post-operative Bohler angle, post-operative Gissane angle, American Orthopedic Foot and Ankle Society (AOFAS) ankle/hindfoot score at long-term follow-up, the delta angle benefit score and the stability of radiological reduction over time. The secondary outcomes included post-operative complications such as infection and osteoarthritis.

Operative technique

**Figure 1:**
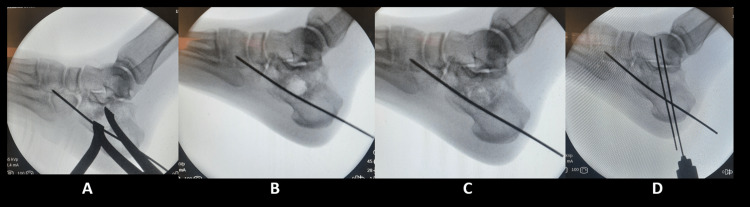
Operative technique images (A) A Meary spreader is introduced through the 2 cm incision into the fracture line between the depressed articular space and the Steinmann pin which would be used as a distractor while elevating the depressed subtalar joint space, (B) reduction of the articular surface, (C) filling the defect with bone allograft, (D) fixation with two additional K-wires

Patients were treated with MIO with BG. This was done through a small 2 cm transverse incision, 1 cm below the lateral malleolus. The lateral sural nerve was retracted superiorly, and the fracture line was identified by blind dissection. A 3.6 mm diameter temporary Steinmann pin was then introduced percutaneously through the posterior calcaneus tuberosity supero-laterally and advanced until seen at the fracture site. Then it was advanced distally under fluoroscopic guidance. The heel was squeezed to reduce the dead space of the calcaneus. A Meary spreader was then introduced through the incision into the fracture line between the depressed articular space and the Steinmann pin which would be used as a distractor while elevating the depressed subtalar joint space. Fluoroscopic guidance (lateral view, Borden’s view, Harris view) was utilized throughout the procedure to assure a good reduction. Once the Bohler angle correction goal was achieved (20°-40°) and length and height were reduced, the reduction was secured by two or three ascending calcaneo-tarsal Steinmann pins. The void created within the calcaneus was filled with a structural freeze-dried femoral head allograft (BioBANK®, a biomedical database). The post-operative protocol included 10-days of posterior below knee splint and non-weight bearing for six weeks. Progressive weight bearing with crutches was allowed if early callus formation was seen on six-week radiographs. Pins were removed at 12 weeks if a union was achieved (Figure 1).

Statistical analysis

Data were analyzed using SPSS software, version 26.0 (IBM Corp., Armonk, NY). Continuous and categorical variables were described as mean and standard deviation and frequency and percentages, respectively. Graphical quantile plots were used to evaluate the normality of continuous variables. Associations between categorical variables were evaluated using the Chi-square test and Fisher’s exact test. Independent samples T-test was used to evaluate differences between two combined Saunders groups (I+II vs III+IV) with the other variables. All reported p values refer to 2-sided tests, and statistical significance was set below an alpha of 5%.

## Results

Demographic characteristics

Twenty-four patients were included in this study (Table 1). Twelve patients had Sanders II fractures (group A) and 12 had Sanders III + IV fractures (group B). The mean follow-up duration was 37.5 months (three-year). The mean age at presentation was 54.23 ± 12.48 (52.36 for group A and 56 for group B). Males were mostly affected (22 out of 24). The mean body mass index (BMI) was 26.10. The most prevalent mechanisms of injury were a fall from height in 20 cases and motor vehicle accidents in four cases. The Bohler angle was diminished in all patients with a mean of 6.04°. It is more affected in group B (mean 0.33°). The mean Gissan angle preoperatively was 139.5° (standard deviation {SD} 20.37). The skin condition at presentation was poor (blood-filled blisters) in 25% of patients equally distributed between the two groups. The mean time to surgery was 5.6 days and the mean operative time was 40 minutes (range from 25 to 60 minutes).

**Table 1:** Demographic characteristics of the study population

Characteristics		Group A (Sanders II)	Group B (Sanders III + IV)	All patients	p-value
Number of Patients		12	12	24	
Follow-up	Mean ± Std.	34.33 ± 21.08	40.67 ± 21.67	37.5 ± 21.15	
Age	Mean ± Std.	52.36±8.12	56.09±15.92	54.23 ± 12.48	0.497
Sex	F	0	2	2	0.476
	M	12	10	22	
Side	Left	6	4	10	0.305
		60.00%	40.00%	100.0%	
	Right	6	8	14	
		42.85%	57.14%	100.0%	
BMI		27.38	24.83	26.10	0.091
		3.88	3.16	3.70	
Operative Delay (days)		3.54	4.12	3.87	0.192
Bohler pre-operative	Mean ± Std.	11.75 ± 24.3	0.33 ± 17.96	6.04 ±21/69	0.158
Gissan pre-operative	Mean ± Std	136.17 ± 19.1	143.00 ± 21.8	139.58 ±20.37	0.423

Radiological results

**Figure 2:**
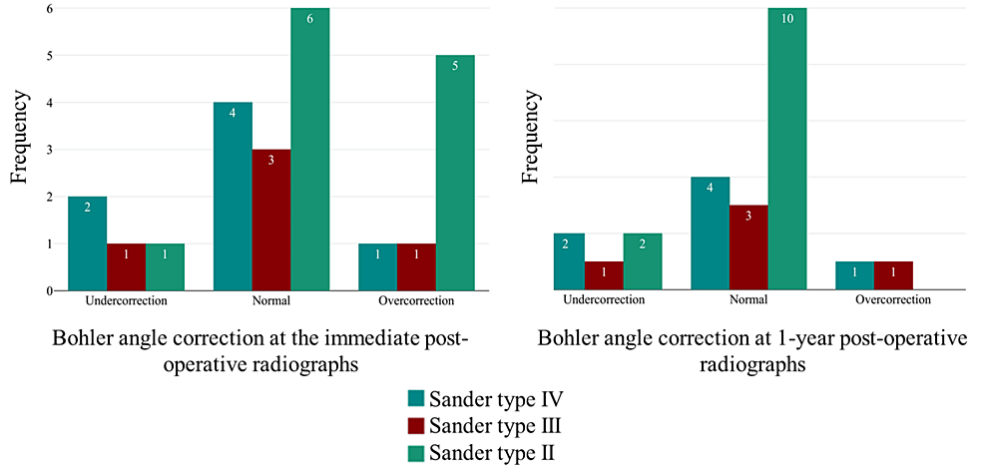
Bohler angle correction by Sanders classification immediately post-operatively and at one-year post-operatively

The radiological results showed significant improvement of Bohler angle from 6.09° ±21,69 preoperatively to 31.79°± 14.1 postoperatively with a p-value<0.001. Group A patients tended to have higher postoperative angles (mean of 35.25 ± 15) with overreduction detected in five patients, compared to a mean of 28.33 ± 12.8 in group B which includes three patients with overreduction. This difference was not statistically significant. Bohler angle varied over time in group A, and radiological results showed a 10° decrease in Bohler angle at one-year follow-up with a p=0.716. On the contrary, group B showed relative stability of the reduction (p=0.460) (Table 2). All group A patients with overreduction had normalization of their Bohler angle at one-year postoperatively (Figure 2). There is a trend to normalization of overcorrected fracture especially Sanders II with a mean reduction of 12,71° ± 11,88 at one-year postoperatively (p=0.05). Gissan angle decreased from 139.5 to 132.5 in the immediate postoperatively.

**Table 2:** Radiological results of minimally invasive osteosynthesis associated with femoral head bone graft in the treatment of intraarticular calcaneus fracture

		Group A (Sanders II)	Group B (Sanders III + IV)	All patients	p-value
Bohler Angle	Preop	11.75 ± 24.3	0.33 ± 17.96	6.04 ±21.69	0.158
	Postop	35.25±15	28.33±12.8	31.79±14.1	0.239
	Delta	20.5	28	25.75	
	p-value	0.021	<0.001	<0.001	
	1-year post-op	24.73±11.24	27.00±15.74	25.87±13.51	
	Delta	10.52	1.33	5.82	
	p-value	0.716	0.469	0.765	
Gissane Angle	Preop	136.17 ± 19.1	143.00 ± 21.8	139.58 ±20.37	0.423
	Postop	130.17 ± 13.01	135 ± 13.09	132.58 ± 13	0.374
	Delta	6	8	7	
	p-value	0.427	0.342	0.201	

Functional results

Concerning functional results, the AOFAS score at the last follow-up (37 weeks ± 21,15) showed 20.83% poor results (AOFAS<50), 50% fair results (AOFAS between 51-74), 16.67% good results (AOFAS 75-94), and 12.5% excellent results (AOFAS>95) without difference between the two groups (p=0.856). The rate of patient satisfaction was 83.3% (45.8% are partially satisfied, and 37.5% are fully satisfied).

Superficial infections (wound inflammation and pin tract infection) were more prevalent in group B (40%) compared to group A (0%) with p=0.014. All of them were treated medically and did not necessitate surgical intervention. Osteoarthritis was frequently encountered in 95.8% of patients at three-year follow-up. Hindfoot varus deformity was found in 58.3% of patients. There were no deep infections in this series (Table 3).

**Table 3:** Complications of the utilized technique during the follow-up period (mean 37.5 weeks)

Complications		Group A (Sanders I + II)	Group B (Sanders III + IV)	All patients	p-value
Superficial infection not necessitating surgical intervention	Incidence	0/12	5/12	5/24	0.014
	Percentage:	0.0%	40.0%	20.8%	
Osteoarthritis	Incidence	11/12	12/12	23/24	1
	Percentage:	91.1%	100%	95.8%	
Hindfoot Varus deformity	Incidence	6/12	8/12	14/24	0.2
	Percentage:	50%	66.67%	58.3%	

## Discussion

In 1952, Essex-Lopresti was one of the first to describe a percutaneous reduction maneuver to deal with the dilemma of IACF management [9]. He provided a detailed technique to lift the depressed articular surface and reduced IACF in a series of 173 patients. He reported good results in 61% of cases based on his assessment of patients' statements and their subjective response to examination. In addition, the return to work was possible in all patients younger than 50 years old. This technique has gained popularity nowadays due to the high prevalence of soft tissue complications with the use of the open technique [7]. Therefore, several authors try to ameliorate this technique using screws, bioabsorbable screws, arthroscopically assisted reduction, balloon reduction, and external fixation to aid reduction [10-15].

Our results demonstrated that the traditional percutaneous technique using K-wires leads to good radiological results, where adequate reduction is achievable in 54.16% of patients on the immediate postoperative radiographs (with a mean corrected angle of 31.79 ± 14.1 (p<.001). This percentage increases to 70.8% at one-year postoperatively. In fact, all Sander type II fractures that were overcorrected initially had a trend to normalize with a delta difference of around 10° and a means sanders II corrected the angle of 24.73° ± 11.24 and a total mean of 25.87° ± 13.51. When we did subgroup analysis, the trend to normalization presents a p=0.05, however, it lacks statistical power due to a low number of patients who were overcorrected. In 2006, Walde et al. published the results of the same technique in a similar population. They achieved a reduction in 70.1% of patients, with a rate of satisfaction as high as 77.7% and a complication rate of 6.5% [16]. They concluded that the MIO technique leads to comparable results and relatively lower complication rates when compared with the open technique.

Despite reporting a relatively fair AOFAS score of 50% in our series, the satisfaction rate shown in our data (83.3%) is comparable to other studies: 77.7% for Walde et al., [16] and 73.9% for Stulik et al. [17]. Shih et al. reported, however, that a good to excellent AOFAS score results in more than 80% of their 20 patients series being treated by percutaneous technique augmented with calcium sulfate cement [18].

Traditional MIO with BG demonstrates low soft tissue complication rates, where no deep infections or wound dehiscence problems were reported, in parallel with low rates of superficial infections (five patients) such as pin tract infections and wound erythema that responded well to antibiotic therapy alone. This data is consistent with similar studies published in the literature, wherein Arastu et al. reported no deep infection and a single superficial infection in their series of 31 patients [19]. Similarly, Shih et al. reported a low infection rate. In contrast, studies that used the open technique demonstrated higher rates of complications with deep infections approaching 14.2% of patients [20]. Our data demonstrate high rates of varus loss of reduction of the hindfoot leading to hindfoot varus deformity in 58.3% of patients. This is a major inconvenience of the technique; patients with this deformity were treated with adaptative soles.

Our study is limited by the low number of patients and its retrospective nature. It provides new data about the long-term radiological results of MIO with BG for joint depression IACF, especially concerning the tendency of overcorrected fracture to normalize at one-year follow-up. Our data insists on the fact that the percutaneous technique has a lower rate of wound complications with comparable functional results.

## Conclusions

MIO as a treatment of IACF when used in association with femoral head allograft gives fair to good functional results. Radiological data demonstrated that when Bohler angle is overreduced >40°, there was a tendency to autocorrection over time. This may be due to progressive depression of the angle over time as weight bearing is authorized; however, this must be analyzed carefully due to the low number of patients who were overreduced (seven patients). Our study demonstrates that this technique carries a high long-term complication rate such osteoarthritis and hindfoot varus despite presenting low soft tissue problem in the early post operative period.
